# Mammographic Breast Density at Breast Cancer Diagnosis and Breast Cancer-Specific Survival

**DOI:** 10.3390/diagnostics14212382

**Published:** 2024-10-25

**Authors:** Ibrahem Kanbayti, Judith Akwo, Akwa Erim, Ekaete Ukpong, Ernest Ekpo

**Affiliations:** 1 Radiologic Sciences Department, Faculty of Applied Medical Sciences, King Abdul-Aziz University, Jeddah 22252, Saudi Arabia; ikanbayti@kau.edu.sa; 2 Medical Image Optimisation and Perception Group (MIOPeG), Faculty of Medicine and Health, The University of Sydney, Camperdown, NSW 2050, Australia; jakw2899@uni.sydney.edu.au; 3 Department of Radiological Sciences, University of Calabar, Calabar 1115, Nigeria; erimakwa@unical.edu.ng (A.E.); ekaeteukpong@unical.edu.ng (E.U.); 4 Department of Imaging and Radiation Therapy, Brookfield Health Sciences Complex, College Road, University College Cork, T12 AK54 Cork, Ireland

**Keywords:** breast cancer, breast density, mammographic density, cancer-specific survival

## Abstract

**Background:** Breast density impacts upon breast cancer risk and recurrence, but its influence on breast cancer-specific survival is unclear. This study examines the influence of mammographic breast density (MBD) at diagnosis on breast cancer-specific survival. **Methods:** The data of 224 patients diagnosed with breast cancer were analyzed. Two area-based MBD measurement tools—AutoDensity and LIBRA—were used to measure MBD via a mammogram of the contralateral breast acquired at the time of diagnosis. These patients were split into two groups based on their percent breast density (PBD): high (PBD ≥ 20%) versus low (PBD < 20%). Breast cancer-specific survival in each of these PBD groups was assessed at a median follow-up of 34 months using Kaplan–Meier analysis and the Cox proportional hazards model. **Results:** The proportion of women with low PBD who died from breast cancer was significantly higher than that seen with high PBD (*p* = 0.01). The 5-year breast cancer-specific survival was poorer among women with low PBD than those with high PBD (0.348; 95% CI: 0.13–0.94) vs. 0.87; 95% CI: (0.8–0.96); *p* < 0.001)]. Women with higher breast density demonstrated longer survival regardless of the method of PBD measurement: LIBRA [log-rank test (Mantel–Cox): 9.4; *p* = 0.002)]; AutoDensity [log-rank test (Mantel–Cox) 7.6; *p* = 0.006]. Multivariate analysis also demonstrated that there was a higher risk of breast cancer-related deaths in women with low PBD (adjusted HR: 5.167; 95% CI: 1.974–13.521; *p* = 0.001). **Conclusion:** Women with <20% breast density at breast cancer diagnosis demonstrate poor survival regarding the disease. The impact of breast density on survival is not influenced by the method of measurement.

## 1. Introduction

Breast cancer (BC) is a public health issue, with one in eight women expected to develop the disease before 80 years of age. One in six women affected by breast cancer die from the disease [1,2]. Post-treatment events, such as recurrence and secondary cancer, are the major determinants of BC-related deaths [3,4]. Identifying and mitigating the risk of these adverse events is crucial to reducing breast cancer-related morbidity and mortality. Clinical models have been established for assessing breast cancer treatment outcomes, including response, survival, and adverse events such as recurrent, secondary, and contralateral BC. These models can be grouped into two categories. The first includes treatment response-monitoring approaches, which include clinical examination, radiological imaging, and pathological evaluation, and the second includes treatment outcome prediction models, which are based solely on clinical and pathologic data [5,6,7]. Each method has significant limitations. All treatment outcome prediction models displayed at best only moderate (AUC ≤ 0.77) discriminatory power—the ability to correctly classify women who will achieve longer survival from those who will not [5,6,7]. Clinical–pathological models are subjective and demonstrate low sensitivity and sampling errors [8,9]. Also, only a few of these models consider recurrence, which is a determinant of breast cancer death. These limitations highlight the need to identify additional markers of breast cancer outcomes to improve the discriminatory power of these models.

Mammographic breast density (MBD), the relative proportion of fibroglandular and fatty tissues within the breast, influences the risk of breast cancer, contralateral breast cancer, and breast cancer recurrence [10,11]. The risk of breast cancer due to breast density has led to the addition of breast density information into breast cancer prediction models and this has improved the discriminatory powers of these models [12,13]. Markers of breast cancer survival, such as estrogen receptors (ERs), progesterone receptor (PRs), and Ki67 status, are also linked to a high MBD [14]. In addition, cancers within dense regions of the breast demonstrate aggressive characteristics such as larger sizes, nodal involvement, and higher histological grades [10,15]. Importantly, a high MBD is associated with the risk of metastasis and recurrence [10,16] and increases the extracellular matrix, which influences drug delivery to the cancer. As such, MBD affects the key determinants of breast cancer deaths [17,18]. These important relationships and interactions suggest that MBD may influence the signaling pathways for markers of breast cancer survival or cause adverse events relating to breast cancer that increase the risk of death from the disease. However, outcome prediction models do not consider the composition of the breast. Therefore, establishing the impact of MBD on breast cancer-specific survival may provide opportunities to improve current outcome prediction models. Also, for breast density to be considered in outcome prediction models, MBD thresholds must be determined to predict breast cancer outcomes, and robust and reproducible methods for establishing these thresholds must be developed.

Only a few studies have explored the influence of MBD on breast cancer-specific survival [19,20,21]. Others have focused on the influence of breast density on cancer-related deaths [22,23,24,25] or examined differences in survival rates between interval and screen-detected cancers [26], exercises which do not consider the time until death or the interval between diagnosis and death. The three studies on survival generated mixed outcomes: they showed that high breast density is associated with poor survival [21], has no association with poor survival [20], or improves survival [19]. It is unclear whether these differences were due to population characteristics and the methods of breast density assessment or differences in the MBD thresholds used to assess breast cancer-specific survival. Therefore, reproducible and quantifiable measures of breast density are needed to establish the influence of MBD on breast cancer-specific survival; however, this has not been explored. It is also unclear whether the discordant findings related to survival were influenced by ethnicity. Therefore, this study examines the influence of MBD on breast cancer-specific survival using two different automated quantitative MBD measurement software packages.

## 2. Materials and Methods

### 2.1. Study Population

**Figure 1:**
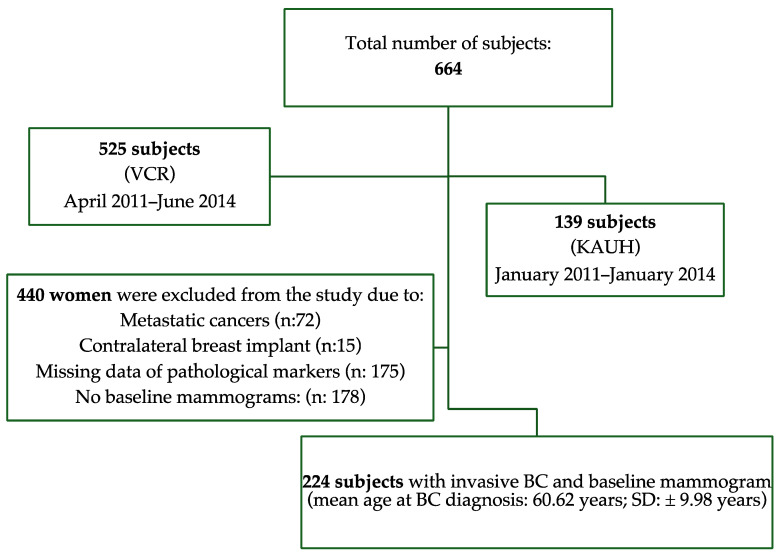
Flowchart of inclusion and exclusion criteria for study.

A retrospective analysis of data from two countries (Australia and Saudi Arabia) was undertaken. Data from 664 women diagnosed with breast cancer who were followed up for six years were retrieved (Saudi Arabia: *n* = 139; Australia: *n* = 525) from hospital databases and cancer registries. Subjects were considered eligible for inclusion in this study if they had a diagnosis of locally invasive breast cancer and if there were mammograms available at breast cancer diagnosis to allow for the quantitative measurement of MBD. Exclusion criteria included women with metastatic breast cancers (*n* = 72); women with contralateral breast implants (*n* = 15), thus avoiding bias in MBD measurement; and women missing data on the clinical and pathological characteristics of the cancer (*n* = 175). Women who did not have baseline mammograms (mammograms at breast cancer diagnosis: *n* = 178) were also excluded. The final cohort comprised 224 women, aged from 26 to 78 years (Mean: 60.62 ± 9.98 years). A flowchart of inclusion and exclusion criteria is shown in Figure 1. The population and disease characteristics of the excluded women were not different from those of the final cohort included in the study (*p* ≥ 0.22). The institutional review boards (IRBs) of King Abdul-Aziz University Hospital (IRB: 449-18) and the University of Sydney (IRB: 2019/459) approved this study and waived patient consent.

### 2.2. Follow Up, Demographic and Clinicopathologic Data

Demographic and clinicopathologic data were retrospectively collected from medical and pathological records and from the breast cancer registry. For each of the women, we collected demographic and anthropometric data such as ethnicity, body mass index (BMI), age at breast cancer diagnosis, and menopausal status. We also collected pathology data such as tumor size, tumor invasiveness, hormone receptor status, histological grade (Nottingham system), and the mode of detection (screen-detected vs. interval cancer detection). Information about the survival status (deceased or survived) and the cause of death was obtained from the Australian registry of deaths (Australian subjects) and from medical records (Saudi subjects). Breast cancer-specific survival was determined from the date of diagnosis to the date of breast cancer-related death. Patients who were alive were censored at their last follow-up or at the study’s endpoint. The median follow-up period was 34 months (interquartile range [IQR]: 28–48 months).

### 2.3. Mammographic Density Measurements

The Laboratory for Individualized Radiodensity Assessment (LIBRA) version 1.0.4 and AutoDensity (version 1.7) software packages were used to independently measure MBD using the craniocaudal projection of contralateral breast diagnostic mammograms. Both LIBRA and AutoDensity are fully automated software packages that use statistical algorithms to segment the breast into dense and non-dense regions during mammograms. The pixel information from these regions is used to estimate percent breast density (PBD). All mammograms of the Saudi population were performed using LIBRA software. Due to incompatibility with computed radiography systems, LIBRA was unable to measure MBD in 27.7% of mammograms from the Australian population. Consequently, the AutoDensity software package was used to measure the MBD in these cases. A reproducibility analysis of 50 cases showed that the MBD measures of LIBRA and AutoDensity were similar (interclass correlation coefficient: 0.95; 95% CI: 0.92–0.96), suggesting that these tools can reproduce their MBD measures and that the measurements of these tools can be combined during analysis. Published breast density studies based on LIBRA and area-based methods reported that population-level PBD distributions were predominantly below 70% and that the mean PBD was within 20% [27,28]. Given these findings and the mean baseline PBD of 19.93% (SD: ±15.84) in our sample, PBD was categorized into low (PBD < 20%)- and high (PBD ≥ 20%)-density groups.

### 2.4. Statistical Analysis

The distribution of MBD was analyzed, using chi-square tests for categorical variables and t-tests or ANOVA for continuous variables. Kaplan–Meier analysis was conducted to estimate breast cancer-specific survival in the low- versus high-PBD groups. Log-rank tests were used to compare the survival rate differences between these groups. The five-year breast cancer survival rates at 95% confidence intervals were determined for all PBD categories [low (PBD < 20%)- and high (PBD ≥ 20%)-density groups]. To identify independent predictors of survival, Cox proportional hazards models were built using backward stepwise regression. The potential interaction between ethnicity and mammographic breast density was explored using Cox proportional hazards regression. Kaplan–Meier analyses were conducted separately with PBD measures of LIBRA and AutoDensity to establish breast cancer-specific survival based on each of these MBD measures. All analyses were performed using IBM SPSS v25 and R v4.0.3, and a two-sided *p* < 0.05 was considered to be statistically significant.

## 3. Results

A total of 224 patients with primary breast cancer were included in this study. Table 1 summarizes the characteristics of the women included in the study. Older, postmenopausal women were more likely to have a low baseline mammographic density (low PBD) compared to younger, premenopausal women (*p* < 0.001). European or Oceanian women demonstrated predominantly low PBD (*p* < 0.001) values and were more likely to die from breast cancer than all other ethnic groups (*p* = 0.01). Women with lower BMI values at breast cancer diagnosis demonstrated higher PBD (*p* = 0.01), carcinoma in situ (*p* = 0.04), and larger tumor size (*p* < 0.05). No significant differences were observed in other tumor characteristics by PBD category (*p* > 0.05).

**Figure 2:**
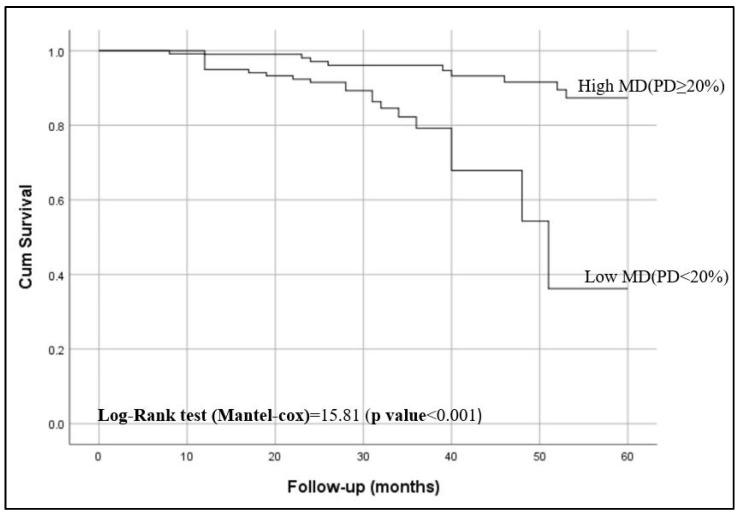
Five-year breast cancer-specific survival of patients according to their percent breast density (PBD): PBD ≥ 20% versus PBD < 20%).

**Figure 3:**
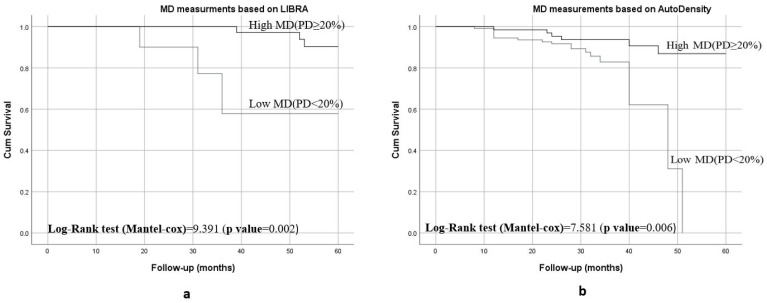
Five-year BC specific survival of patients according to the type of MD assessment methods for PBD ≥ 20% versus PBD < 20%: (**a**) 5-year survival when assessed by LIBRA PBD; (**b**) 5-year survival when assessed by AutoDensity PBD.

Kaplan–Meier curves showed that women with <20% breast density had shorter breast cancer-specific survival compared to those have ≥20% breast density (*p* < 0.001), as shown in Figure 2. Figure 3 shows the 5-year breast cancer-specific survival estimated for PBD ≥ 20% versus PBD < 20% by LIBRA (Figure 3a) and AutoDensity (Figure 3b). When the analyses were nested within the method of breast density measurement (either PBD measures of LIBRA or AutoDensity), women with low PBD consistently demonstrated shorter survival [LIBRA (log-rank test = 9.391, *p* = 0.002); AutoDensity (log-rank test = 7.581, *p* = 0.006)], as shown in Figure 3.

The five-year breast cancer-specific survival rate for women with low PBD was 0.348; 95% CI: 0.13–0.937 (Table 2). Multivariate analysis revealed that women with low PBD were at significantly higher risk of death compared to those with higher PBD (hazard ratio [HR] = 5.737, *p* < 0.001). The risk of death due to low PBD was not attenuated after adjusting for ethnicity (*p* = 0.01), as shown in Table 3. No interaction was observed between ethnicity and PBD.

## 4. Discussion

The data produced demonstrate that women with low percent breast density (PBD < 20%) at breast cancer diagnosis are more likely to die from breast cancer and have poorer breast cancer-specific survival, regardless of their ethnicity. The influence of breast density on deaths and survival rates remained unchanged after stratifying the analysis by the method of mammographic breast density measurement (LIBRA or AutoDensity). There is a paucity of evidence to explain the mechanism linking breast density and breast cancer-related deaths or survival. However, the breast microenvironment has been implicated in poorer survival outcomes for women with low breast density [22]. Dense tissue contains a stronger extracellular matrix (ECM); higher levels of proteoglycans, which regulate the response of ECM to mechanical forces [29]; and higher levels collagen, which supports breast cancer re-organization. A high breast density has also been shown to support metastasis and recurrence [10], increasing the risk of death from breast cancer. On the other hand, the weaker ECM seen in fatty breasts allows for cell migration and drug delivery to cancer cells [30]. Logically, if the relationship between breast cancer-specific survival and breast density was related to the ECM, women with high breast density would demonstrate worse survival than those with fatty breasts.

A plausible explanation for the higher risk of death and poor survival in women with low breast density may be linked to changes in breast composition following treatment and the characteristics of and breast cancer treatment interventions for older women. It has been shown that breast density changes over time and that younger, premenopausal women, with dense breasts at breast cancer diagnosis and normal BMI values, experience longitudinal reductions in breast density [31]. It has also been shown that premenopausal women undergoing tamoxifen therapy experience a decrease in breast density [32], and that women who experience a decrease in breast density following treatment have a reduced risk of death from breast cancer [33,34]. It is possible that the better breast cancer-specific survival in women with ≥20% baseline PBD is linked to longitudinal reductions in breast density following breast cancer treatment compared to women with <20% breast density. It is also possible that the higher risk of death and poorer survival observed in our work and previous pieces of work [22,23,26] may be due to the overrepresentation of older and postmenopausal women in the low-breast-density group.

To ensure that our findings can be explained, we considered breast cancer prognostic factors such as tumor size, invasiveness, HER2, PR, and ER status, and tumor grade. We also examined other factors that influence deaths from breast cancer such as the mode of detection, the age at breast cancer diagnosis, postmenopausal status, and BMI. We did not find any difference between the low- and high-PBD groups in terms of the mode of detection, HER2, PR, and ER status, and tumor grade. While the mean tumor size was higher in the higher PBD group, tumor invasiveness, which is a stronger independent prognostic factor for breast cancer [35], was very common in the low-PBD group. Also, women in the low-PBD group were significantly older at breast cancer diagnosis, postmenopausal, and overweight or obese (see Table 1). It is well known that obesity is associated with a 35–40% increased risk of death from breast cancer and poorer disease-specific survival [36], and that postmenopausal women have the highest BC-related mortality rates (25.5 per 100,000 women) [37]. We believe that the combined effects of tumor invasiveness, age at breast cancer diagnosis, postmenopausal status, and having overweight or obesity may contribute to the poorer survival outcomes for women in the low-PBD group. Therefore, the poorer BC-specific survival in women with low PBD may be influenced by the characteristics of the population demonstrating low PBD. Breast cancer is a heterogenous disease, requiring treatment to be tailored to its characteristics. Older postmenopausal women who often receive adjuvant raloxifene, tibolone, and aromatase inhibitors generally demonstrate poorer disease-specific survival [38] compared to premenopausal women, who often receive adjuvant tamoxifen. It can be argued that the differences in primary or adjuvant treatments received by the women included in this study may impact the outcome; however, it should be remembered that whatever primary or adjuvant treatments these women received were considered the best choice of treatment by their oncologists. Unfortunately, the small number of death events, premenopausal and younger women in our sample, and treatment data limited our ability to stratify the Cox proportional hazards model by menopausal status, age, age at breast cancer diagnosis, and treatment intervention. Therefore, longitudinal studies that account for changes in breast density over time and the confounding effects of age, menopausal status, body mass index, ethnicity, and treatment interventions may better explain the poorer survival outcomes for women with <20% breast density

Studies that examined the influence of breast density on breast cancer-specific survival generated mixed results: high breast density is associated with poor survival [21], MBD does not influence breast cancer-specific survival [20], and women with low breast density demonstrate poor survival [19]. These discordant findings could be attributed to differences in population characteristics and the reliance on the qualitative classification of breast density. The BI-RADS atlas and the Boyd’s six-category classification scale used in these previous studies [19,20,21] are observer-dependent and are limited by inter-user variability [39,40]. These methods also do not consider the thickness of the dense tissue in the dense area which may not accurately capture the amount of fibroglandular tissue in the dense area [39,40]. There were also differences in the MBD thresholds used to assess breast cancer-specific survival. In one study [19], women with <25% density were considered very low breast density. In one study, survival was assessed for each BI-RADS breast density category [20], while another study stratified breast density according to three levels: fatty (BI-RADS 1), moderate (BI-RADS 2 + 3), or dense (BI-RADS 4) [21]. The thresholds used in two of these studies [20,21] may have limited their ability to identify the influence of breast density on survival. These differences in thresholds impact upon the findings reported in the literature and make the comparison of results difficult. Using a two-category MBD classification [dense (BI-RADS 3–4) versus non-dense (BI-RADS 1–2)], as implemented in studies examining the effect of breast density on breast cancer risk or mammographic sensitivity, would have allowed us to more correctly capture the influence of breast density on breast cancer-specific survival. The six-category classification scale used in one study [20] has density values up to 100%, whereas the highest MBD reported by quantitative volumetric methods is under 50% [41].

Published evidence from a different study, conducted using semiautomated tools, shows a mean PBD of 18.5% [31] and similar breast density results for the right and left breasts [42]. In the current study, we relied on two automated and reproducible methods of breast density assessment—LIBRA and AutoDensity—for the contralateral breast and demonstrated a mean PBD of 19.9%, with the highest values recorded for extremely dense breasts being under 70% [27,28]. This mean PBD and the range reported in the literature for area-based methods [26,27,28,31] informed our use of the 20% cut-off. Regardless of the method used for PBD assessment, women with <20% PBD consistently demonstrated poorer survival. Whilst our thresholds for discriminating between high and low PBD align with published evidence from the assessment of risk in relation to breast density and longitudinal changes in breast density [31], these thresholds may not completely reflect thresholds for the classification of women into low- and high-breast-density categories in clinical practice [40]. Area-based quantitative measures of breast density are currently used for research and epidemiology, and thresholds have not been established to represent the clinical low- and high-breast-density categories defined in the widely used BI-RADS Atlas. Therefore, caution should be exercised when extrapolating our breast density thresholds to the BI-RADS Atlas or clinically used breast density categories. Validation studies are needed to establish the best thresholds for discriminating between high and low MBD using quantitative area-based tools.

Other factors that may have contributed to the discordant findings in the literature may include the characteristics of the cancers included in the analyses, the methods used to establish survival, and the lack of adjustments for important confounders. One of these studies assessed the deaths of some women based on the presumption that those with metastatic cancer may have died from the disease, albeit without confirming their survival status or period, and studied women with local and metastatic disease [21], which may have influenced the findings reported. Another study combined both screen-detected and interval cancers in the analysis based on different tiers of breast density [20], which could have masked the effect of breast density on breast cancer-specific survival. When their analysis was nested on screen-detected cancers, survival rates were slightly higher among women with heterogeneously dense and extremely dense breasts, but when screen-detected and interval cancers were combined, this effect was reversed, although it did not reach statistical significance [20]. We excluded women with interval cancer and metastatic cancer to avoid the bias these may introduce to the analysis, adjusted for factors that influence MBD, and showed that features such as tumor size, histological grade, and molecular characteristics do not obscure the influence of MBD on breast cancer-specific poor survival. Therefore, our findings were not influenced by tumor characteristics. We also included women of different races to ensure that the findings represent the influence of breast density on breast cancer-specific survival. Thus, the findings have two clinical implications: first, they suggest that the incorporation of MBD at the time of breast cancer diagnosis in breast cancer prognostic models may improve their discriminatory powers. Secondly, within the context of breast screening, the findings can inform health care practice and policies aimed at the treatment of non-dense postmenopausal women to improve disease-specific survival.

The limitations of this study include the small number of mortality events in the different MBD categories, which resulted in wide confidence intervals. This small number of events also limited our ability to explore the modifying effects of menopausal status and age at breast cancer diagnosis on the influence of MBD on breast cancer-specific survival. This small number of mortality events is reasonable because widespread population screening and improvements in treatment strategies have significantly reduced mortality rates. Like in previous studies, most of the women included in our sample were older and postmenopausal; this is expected, as many screening programs target perimenopausal and menopausal women. Nonetheless, our study included racially diverse populations, was based on MBD at the time of diagnosis, and used two fully automated and reproducible tools to measure MBD. The similar outcomes for LIBRA and AutoDensity PBD measurements suggest that our results can be relied upon to establish the influence of MBD on breast cancer-related deaths and survival.

## 5. Conclusions

Women with a low percent mammographic breast density at breast cancer diagnosis demonstrate poor survival regarding breast cancer. Automated tools can reproduce the influence of mammographic breast density on breast cancer-specific survival. Factors such as tumor invasiveness, age at breast cancer diagnosis, postmenopausal status, and having overweight or obesity may explain the poorer survival outcomes in women exhibiting <20% breast density.

## Figures and Tables

**Table 1:** Patient characteristics and cancer features according to PBD categories.

Patients Characteristics and Cancer Features		All Patients *n* (%)	PBD (20% Cut-off)		*p* Value
			PBD < 20%	PBD ≥ 20%	
Number of deaths: *n* (%)	Censored	190 (84.8)	94 (79)	96 (91.4)	**0.01**
	Deaths	34 (15.2)	25 (21)	9 (8.6)	
Age at BC diagnosis: *n* (%)	Age < 50 years	23 (10)	2 (8.7)	21 (91.3)	**<0.001**
	Age ≥ 50 years	201 (90)	117 (58.2)	84 (41.8)	
Age at BC diagnosis: mean (95% CI) (years)			64.48 (63.13, 65.83)	56.12 (54.05, 58.20)	**<0.001**
Ethnicity: *n* (%)	European	55 (24.55)	34 (61.8)	21 (38.2)	**<0.001**
	Oceanian (Australians and New Zealanders)	109 (48.66)	70 (64.2)	39 (35.8)	
	Asian	6 (2.67)	2 (33.3)	4 (66.7)	
	Arab	47 (20.98)	9 (19.1)	38 (80.9)	
	African	2 (0.89)	1 (50)	1 (50)	
	Jewish	2 (0.89)	1 (50)	1 (50)	
Menopause status: *n* (%)	Postmenopausal	201 (90)	116 (57.7)	85 (42.3)	**<0.001**
	Premenopausal	23 (10)	3 (13)	20 (87)	
BMI: *n* (%)	Underweight (<18.5 kg/m^2^)	4 (1.78)	2 (50)	2 (50)	**0.01**
	Normal (18.5–24.9 kg/m^2^)	84 (37.5)	33 (39.3)	51 (60.7)	
	Overweight (25–29.9 kg/m^2^)	73 (32.58)	43 (58.9)	30 (41.1)	
	Obese (≥30 kg/m^2^)	47 (20.98)	31 (66)	16 (34)	
Mode of detection: n (%)	Screen detected	92 (41.07)	58 (63)	34 (37)	0.19
	Interval cancers	32 (14.28)	16 (50)	16 (50)	
ER status: *n* (%)	Positive	149 (66.51)	74 (49.7)	75 (50.3)	0.58
	Negative	29 (12.94)	16 (55.2)	13 (44.8)	
PR status: *n* (%)	Positive	131 (58.48)	64 (48.9)	67 (51.1)	0.52
	Negative	46 (20.53)	25 (54.3)	21 (45.7)	
HER2 status: *n* (%)	Positive	31 (13.83)	13 (41.9)	18 (58.1)	0.25
	Negative	141 (62.94)	75 (53.2)	66 (46.8)	
Tumour invasiveness: *n* (%)	IC	172 (76.78)	90 (52.3)	82 (47.7)	**0.04**
	in situ	7 (3.12)	1 (14.3)	6 (85.7)	
Grade: *n* (%)	Low	28 (12.5)	12 (42.9)	16 (57.1)	0.35
	Intermediate	81 (36.16)	40 (49.9)	41 (50.6)	
	High	62 (27.67)	36 (58.1)	26 (41.9)	
Tumour size: *n* (%)	Less than 1 cm	55 (24.55)	31 (56.4)	24 (43.6)	0.38
	1–2 cm	48 (21.42)	21 (43.8)	27 (56.3)	
	More than 2 cm	65 (29.01)	30 (46.2)	35 (53.8)	
Tumour size: Mean (95% CI) (cm)			0.643 (0.453, 0.865)	1.18 (0.864, 1.563)	**0.005**

Abbreviations: 95% CI—95% confidence interval; BMI—body mass index; IC—invasive cancers; in situ—in situ cancers.

**Table 2:** Five-year breast cancer-specific survival.

PBD Categories	5 Years Survival Rate	95% Confidence Interval	*p* Value
PBD (20% Cut-off)			
PBD < 20%	0.348	(0.130, 0.937)	<0.001
PBD ≥ 20%	0.873	(0.795, 0.959)	

**Notes:** PBD: percent breast density; *p* values were based log-rank test.

**Table 3:** Unadjusted and adjusted Cox proportional hazards ratios of death.

PBD Measures	Deaths	Unadjusted HR	*p* Value	Adjusted HR	*p* Value
PBD (20% Cut-off)					
PBD ≥ 20%	9	**Reference**		**Reference**	
PBD < 20%	20	5.737 (2.292–14.362)	<0.001	5.167 (1.974–13.521)	0.001 ^a^

Abbreviations: HR—hazard ratio; PBD—percent breast density; ^a^—adjusted for ethnicity.

## Data Availability

Data for the Australian subjects included in the study are available upon the approval of the Data custodian and the institutional review board. However, due to privacy and ethical restrictions, data from the Saudi subjects are not available.
